# From Formative Research to Cultural Adaptation of a Face-to-Face and Internet-Based Cognitive-Behavioural Intervention for Arabic-Speaking Refugees in Germany

**DOI:** 10.32872/cpe.4623

**Published:** 2021-11-23

**Authors:** Maria Böttche, Christina Kampisiou, Nadine Stammel, Rayan El-Haj-Mohamad, Carina Heeke, Sebastian Burchert, Eva Heim, Birgit Wagner, Babette Renneberg, Johanna Böttcher, Heide Glaesmer, Euphrosyne Gouzoulis-Mayfrank, Jürgen Zielasek, Alexander Konnopka, Laura Murray, Christine Knaevelsrud

**Affiliations:** 1 Clinical-Psychological Intervention, Freie Universität Berlin, Berlin, Germany; 2 Center Überleben, Berlin, Germany; 3 Department of Psychology, University of Zurich, Zurich, Switzerland; 4 Institute of Psychology, University of Lausanne, Lausanne, Switzerland; 5 Clinical Psychology & Psychotherapy, Medical School Berlin, Berlin, Germany; 6 Clinical Psychology and Psychotherapy, Freie Universität Berlin, Berlin, Germany; 7 Clinical Psychology and Psychotherapy, Psychologische Hochschule Berlin, Berlin, Germany; 8 Medical Psychology and Medical Sociology, University of Leipzig, Leipzig, Germany; 9 LVR-Institute for Healthcare Research, Cologne, Germany; 10 Health Economics and Health Services Research, University Medical Center Hamburg-Eppendorf, Hamburg, Germany; 11 Johns Hopkins University, Baltimore, MD, USA; Philipps-University of Marburg, Marburg, Germany

**Keywords:** cultural adaptation, transdiagnostic, refugees, decision-making process

## Abstract

**Background:**

This study aims to provide a transparent and replicable documentation approach for the cultural adaptation of a cognitive-behavioural transdiagnostic intervention (Common Elements Treatment Approach, CETA) for Arabic-speaking refugees with common mental disorders in Germany.

**Method:**

A mixed-methods approach was used, including literature review, interviews, expert decisions and questionnaires, in order to adapt the original CETA as well as an internet-based guided version (eCETA). The process of cultural adaptation was based on a conceptual framework and was facilitated by an adaptation monitoring form as well as guidelines which facilitate the reporting of cultural adaptation in psychological trials (RECAPT).

**Results:**

Consistent with this form and the guidelines, the decision-making process of adaptation proved to be coherent and stringent. All specific CETA treatment components seem to be suitable for the treatment of Arabic-speaking refugees in Germany. Adaptations were made to three different elements: 1) Cultural concepts of distress: a culturally appropriate explanatory model of symptoms was added; socially accepted terms for expressing symptoms (for eCETA only) and assessing suicidal ideation were adapted; 2) Treatment components: no adaptations for theoretically/empirically based components of the intervention, two adaptations for elements used by the therapist to engage the patient or implement the intervention (nonspecific elements), seven adaptations for skills implemented during sessions (therapeutic techniques; two for eCETA only) and 3) Treatment delivery: 21 surface adaptations (10 for eCETA only), two eCETA-only adaptations regarding the format.

**Conclusion:**

The conceptual framework and the RECAPT guidelines simplify, standardise and clarify the cultural adaptation process.

## Highlights

- The framework and the guidelines allow for a reproducible and systematic cultural adaptation.
- The flexible and simple format of the original CETA manual requires mainly surface adaptations.
- eCETA requires additional adaptations compared to the face-to-face version.

Arabic-speaking refugees from the MENA (Middle East and North Africa) region have constituted the largest group of refugees in Germany in recent years (Federal Office for Migration and Refugees, 2020). Epidemiological studies on the mental health of asylum seekers and refugees indicate high prevalence rates of mental disorders, especially for posttraumatic stress disorder (PTSD) and depression (Nesterko et al., 2020; Turrini et al., 2017).

Despite the need for psychological treatment among refugees, only a minority utilise specialised mental health care services. Göpffarth and Bauhoff (2017) reported that refugees in Germany have six psychotherapist contacts per 1,000 health-insured persons, compared to 20 contacts for non-refugee persons. Reasons for this treatment gap lie in structural barriers (e.g. difficult to access the health system, post-migration difficulties, regional lack of trained therapists, long waiting lists) and cohort-specific characteristics (e.g. language, fear of stigmatisation, comorbid disorders), but are also due to a general lack of psychotherapeutic treatments for culturally diverse groups (Colucci et al., 2015; Sijbrandij, 2018).

Transdiagnostic approaches seem to be especially promising for the treatment of a wide range of psychological symptoms, as they can be effectively applied for different and comorbid disorders (Newby et al., 2015; Reinholt & Krogh, 2014). A prominent evidence-based transdiagnostic approach for war-torn populations is CETA (Common Elements Treatment Approach; Murray et al., 2014, Supplement 1: modules and content). CETA has proven to be effective in reducing common mental health problems in culturally diverse settings in low- and middle-income countries (e.g. Zambia: Kane et al., 2017; Iraq: Weiss et al., 2015). It addresses symptoms of depression, anxiety, substance use and trauma-related disorders, and follows a tailored approach, i.e. element selection, sequencing and dosage vary depending on symptom presentation. CETA might also be a promising approach to reduce the treatment gap for refugees in European countries.

Additionally, an internet-based format would enable a wider reach, since it does not depend on geography (e.g. lack of trauma therapists in the local area), and communication between client and counsellor can be asynchronous. An internet-based version could also overcome the fear of stigmatisation due to the visual anonymity of the online format. As many refugees in high-income countries use the internet (Gillespie et al., 2016), internet-based interventions are easily accessible for refugee populations.

In order to tailor mental health interventions to the context and needs of diverse cultural groups, there has been an increasing focus on culture-sensitive interventions. Meta-analyses generally indicate a superiority of culturally adapted interventions for the respective target group over non-adapted interventions (Hall et al., 2016; Harper Shehadeh et al., 2016), although it should be noted that most adaptations did not follow a systematic procedure, thus limiting the ability to compare and replicate their findings.

To overcome this weakness, a conceptual framework for cultural adaptation of interventions for common mental disorders was developed (Heim & Kohrt, 2019). This framework consists of three main elements: 1) cultural concepts of distress, including cultural explanations, cultural syndromes, idioms of distress; 2) treatment components, comprising specific and unspecific elements and therapeutic techniques; and 3) treatment delivery including delivery format, surface adaptation and setting. Specific elements refer to interventions that are based on theoretical assumptions, such as behavioural or cognitive approaches; unspecific treatment elements are the common factors such as the therapeutic relationship or providing a meaningful treatment rationale; and therapeutic techniques refer to exercises and other interventions that are undertaken to transmit the therapeutic components, such as role plays or homework (Singla et al., 2017). In addition, the framework by Heim and Kohrt (2019) includes “surface adaptations”, which refer to matching materials and illustrations to the target population (Resnicow et al., 1999). This framework has been extended and translated into a set of reporting criteria for the cultural adaptation of psychological interventions (Heim et al., 2021, this issue; Supplement 2).

In conclusion, some of the existing barriers to psychological treatment provision for refugees in Europe might be addressed by culturally adapted and transdiagnostic interventions with different delivery formats. Thus, the aim of the present study was to conduct a culture-sensitive adaptation of a cognitive-behavioural transdiagnostic intervention (CETA) for Arabic-speaking refugees with common mental health disorders in Germany in a transparent and replicable manner, based on the framework (Heim & Kohrt, 2019) and the guidelines of reporting cultural adaptation in psychological trials (RECAPT, Heim et al., 2021, this issue). The study focuses on the decision-making process, i.e., the process from assessing cultural concepts of distress to adapting treatment components. The adaptation was conducted both for the original face-to-face context and for an internet-based context (eCETA).

## Method

### Procedures and Participants

The process of cultural adaptation in this study used the RECAPT guidelines (Heim et al., 2021, this issue), and consists of six steps (details on the procedures followed and the study participants are presented in Supplement 2, RECAPT guidelines; Supplement 3, adaptation monitoring form, and Supplement 4, COnsolidated criteria for REporting Qualitative research [COREQ] checklist):

First, in a workshop with the CETA developers as well as in discussions of the research team, all interventional components (e.g. treatment components, therapeutic techniques, expressions) were identified in the treatment manual and included in the free list and key informant interviews in Step 3.

Second, a literature review was conducted regarding existing cultural concepts of distress among Arabic-speaking persons in the MENA region (e.g. idioms of distress, cultural explanations).

Third, semi-structured interviews and focus groups were conducted to discuss cultural concepts of distress and treatment components with Arabic-speaking refugees/migrants and mental health experts. Participants included i) Arabic-speaking potential users without a medical and/or psychosocial background (AU, Arabic Users; *n* = 20); ii) Arabic-speaking mental health professionals with a migration and refugee background (AP, Arabic Professionals; *n* = 11); iii) mental health experts working with refugees in different institutions in Germany (HE, Health Experts; *n* = 6). Additionally, two focus groups of Arabic-speaking mental health professionals (male and female) discussed inconsistent results of the interviews (FG, *n* = 7). The structure of the interviews and focus groups was based on Module 1 of the established Manual for Design, Implementation, Monitoring, and Evaluation of Mental Health and Psychosocial Assistance Programs for Trauma Survivors in Low Resource Countries (Applied Mental Health Research Group, 2013). The interviews were carried out on the basis of semi-structured interviews. Each type of interview contains different, non-overlapping closed and open-ended questions. In addition to the interviews, potential users (AU, *n* = 20) and Arabic-speaking professionals (AP, *n* = 11) also completed the “Barts Explanatory Model Inventory-Checklist” (BEMI-C), which assesses cultural concepts of distress (Rüdell et al., 2009).

Fourth, all adaptations and examinations were listed and summarised in a monitoring form (Supplement 3, Heim et al., 2021, this issue).

Fifth, final agreements on the adapted version were made with the help of four independent Arabic-speaking experts, who evaluated the suggested adaptations based on the aforementioned steps (Supplement 3).

Sixth, any differences between the preliminary adapted version and the experts’ suggestions were discussed within the research team and a final decision was made (Supplement 3).

### Data Collection

After receiving information about the study, participants signed an informed consent form prior to participating in the interviews/focus groups. All forms were provided in Arabic. The interviews (AU, AP) and the FG were conducted by Arabic-speaking trained interviewers. All interviews/FGs were audio-recorded, and the recordings were summarised and translated into German. An interview ID was assigned by the first author, who kept an encrypted digital document with the identifying keys. Basic non-identifying information about the respondents was collected (age, gender).

Interviews were conducted in Berlin and Cottbus, Germany, between December 2019 and May 2020. Final agreements (Step 6 in Procedures and Participants) were made between July and September 2020. All participants (Table 1 for more details) received an incentive for their participation (20-40 Euros). The Ethics Committee of the Freie Universität Berlin (Germany) gave approval for this study (008/2020).

**Table 1:** Sample Description of Participants of the Formative Research (Step 3)

Interviews/Focus Group	Sample size	Age in years *M* (*SD*)	Age range	Sample size	Age in years *M* (*SD*)	Age range
Free list interview (Arabic Users)						
total	20	30.10 (8.86)	23-57			
men	15	27.40 (3.94)	23-39			
women	5	38.20 (14.46)	24-57			
Key informant interview (Arabic Professionals)				Key informant interview (Health Experts)		
total	11	30.78 (5.36)	23-37	6	47.83 (12.81)	32-68
men	5	31.80 (5.63)	23-37	2	45.50 (12.02)	37-54
women	6	29.50 (5.51)	24-36	4	49.00 (14.83)	32-68
Focus Group I				Focus Group II		
men	3	28.33 (4.73)	23-32	–	–	–
women	–	–	–	4	27.75 (3.50)	24-32

*Note.* *SD* = Standard Deviation.

### Data Analysis

The data were analysed using content analysis, i.e. transcripts of the communication were evaluated and prepared for the adaptation process (Rädiker & Kuckartz, 2019) with the help of MAXQDA 2018 (VERBI Software, 2018). All responses from the AU, AP and HE interviews were listed and coded. No prior coding framework existed. A coding system was developed inductively for all three forms of interviews. Codes represented the themes provided in responses to the open-ended questions and were summarised quantitatively (e.g. 18 out of 20 AU interviewees named sport as a positive activity: sport *n* = 18/20). The frequency of the answers can be interpreted as an indicator of their importance (Applied Mental Health Research Group, 2013). Each group received different questions, so the second number always indicates the interview group (*n*/20 = AU, *n*/6 = HE, *n*/11 = AP). All data were analysed at the individual level, with the exception of data from the FGs, which were analysed at the group level. Findings of the FGs aimed to complement or contrast findings from AU, AP and HE interviews. The themes that arose from the coded framework were presented to the four Arabic-speaking experts and finalised by the research team.

Quantitative data from BEMI-C were analysed using the SPSS software, version 26 (IBM Corporation, 2018).

## Results

### Decision-Making and Expert Reviews

For the decision-making process, the monitoring form (Supplement 3) was used. Here, all preliminary and final adaptations in the process were written down and discussed.

First, two one-day workshops of the research group (see Supplement 2) took place in Berlin, Germany, to discuss and evaluate the results of the FL and KI interviews. These results were prepared by MB (first author) and a psychology student (RE). During the workshop, the prepared results and suggestions were read by all participants (written in the monitoring form). There was either agreement with the adaptation or further suggestions were made.

Second, based on the two workshops, the content of the FGs was elaborated and the first version of the adaptations from the workshops was adapted in writing in the document.

Third, based on the results of the FGs, the existing adaptations were modified and written down if necessary.

Fourth, the four Arabic-speaking experts were sent the form with all of the existing preliminary adaptations. The experts either agreed to the proposals in writing or noted changes in writing in the monitoring form. Explicit linguistic comments were also made here.

Finally, another two-day workshop of the research group took place. This was again prepared by MB and RE, who had written down the suggestions of the four experts so that the members of the research group could see the changes beforehand. During these two days, all changes in the document were discussed and voted on.

### Cultural Concepts of Distress (CCD)

Three cultural adaptations of CETA were made with regard to the CCD. Two adaptations were made regarding idioms of distress. First, based on the BEMI-C (AU & AP), five idioms of distress were integrated into the introduction of CETA/eCETA (Table 2; in bold). Second, the AP interviews showed that the assessment of suicidal ideation in the component “Safety” should be carried out gradually, i.e. with the topic being introduced indirectly (*n* = 6/11, e.g. “Have you had thoughts that you would be better off dead or not waking up in the morning?”), followed by direct questions regarding suicidal thoughts and plans.

**Table 2:** Typical Somatic and Mental Symptoms of Arabic-Speaking Refugees (Selection From BEMI-C)

Symptoms	Free List Interview (AU)			Key Informant Interview (AP)		
	Total	Men	Women	Total	Men	Women
Somatic *n* (%)						
**Sleep disturbances**	15 (75)	11 (73.3)	4 (80)	9 (81.8)	4 (80)	5 (83.3)
Pain/aches	17 (85)	12 (80)	5 (100)	10 (90.9)	4 (80)	6 (100)
**Fatigue/tiredness**	20 (100)	15 (100)	5 (100)	11 (100)	5 (100)	6 (100)
**Nerves/being agitated/restless**	19 (95)	14 (93.3)	5 (100)	11 (100)	5 (100)	6 (100)
Bodily weakness	16 (80)	11 (73.3)	5 (100)	9 (81.8)	4 (80)	5 (83.3)
Nausea or feeling sick	12 (60)	7 (46.7)	5 (100)	8 (72.7)	5 (100)	3 (50)
Mental *n* (%)						
Dysphoria (feeling down)	9 (45)	4 (26.7)	5 (100)	6 (54.5)	2 (40)	4 (66.7)
Feeling irritable or fed up/bored	20 (100)	15 (100)	5 (100)	7 (63.6)	3 (60)	4 (66.7)
**Feeling nervous, anxious**	17 (85)	13 (86.7)	4 (80)	9 (81.8)	5 (100)	4 (66.7)
**Feeling frightened or fearful**	17 (85)	12 (80)	5 (100)	9 (81.8)	4 (80)	5 (83.3)
Lack of concentration/forgetfulness	18 (90)	13 (86.7)	5 (100)	10 (90.9)	5 (100)	5 (83.3)
Loss of interest/ not being able to enjoy things	18 (90)	13 (86.7)	5 (100)	7 (72.7)	5 (100)	3 (50)

*Note.* Bold, five most prominent symptoms included in the manual.

The description of Arabic-speaking refugees’ CCD (Hassan et al., 2015), as well as the data from the HE interviews, highlighted the importance of an exploratory model of psychological symptoms. Therefore, the introduction of CETA/eCETA was expanded with a section addressing fear of becoming crazy, the relationship between body and mind, and awareness of mental health problems (HE: *n* = 3/6, Supplement 2).

### Treatment Components (Specific and Unspecific Elements, and Therapeutic Techniques)

With regard to therapeutic treatment components, HE stated that all specific CETA components were suitable for the treatment of Arabic-speaking refugees. Therefore, all components remained in the adapted manual (Supplement 1).

The HE interviews did not result in a clear conclusion regarding the fit of the component “Problem solving”, since half of the respondents assessed the content as not feasible (e.g. too cognitive, difficult to work with). Therefore, the focus groups and the research group discussed this component further during the process. Ultimately, the component remained in the manual, as it was considered useful to address post-migration living difficulties.

Regarding the unspecific treatment elements, two adaptations were carried out in the introductory part of CETA. First, the component “Encouraging Participation” was extended regarding the presentation of the rules of interpretation, because interpreters are of crucial importance in the face-to-face context. Second, the literature and AP interviews (*n* = 11/11) emphasised the importance of understanding the treatment process in order to increase compliance (e.g., patients' active role during sessions, possible destabilisation). Therefore, the analogy of “walking on a mountain path” was explicitly added to this component in CETA/eCETA (Supplement 2).

Based on discussions and practical experiences of the research group, four therapeutic techniques were excluded due to the difficulties in implementation and delivery in an adequate online format (Supplement 1). Results from the AP interviews showed that all other therapeutic techniques were suitable (Supplement 1). Due to the asynchronous communication of lay counsellor and patient in eCETA, two role plays were adapted. This therapeutic technique, which requires simultaneous interaction, was transformed into a written "letter to a friend", in which the patient addresses an imaginary friend with the same problem. Also due to the asynchronous communication, the decision was made to fix the order of the techniques in TDW-II in CETA/eCETA.

### Treatment Delivery (Format, Surface)

Regarding the delivery format, two changes were implemented. First, CETA is also offered in an internet-based context (eCETA). Second, the handling of self-endangering and third-party-endangering behaviour had to be adapted for eCETA, which is conducted by lay counsellors. As soon as such behaviour is detected, the communication immediately changes from asynchronous to synchronous (i.e., telephone).

The final category of adaptations refers to the surface, e.g. text, examples, and migration-, language-, and culture-related material. Arabic-speaking individuals (AU, AP) revealed that the expressions used in the manual are for the most part culturally appropriate. Four specific adaptations were made (i.e. translation of the phrases “a day in the life” and “here and now”, expressions for “suicide” and “suicidal ideation and plans”). All other 17 adaptations are shown in Supplement 5).

## Discussion

In this study, the transdiagnostic CETA was adapted for Arabic-speaking refugees in Germany. The cultural adaptation process followed an approach that enables a replicable and systematic documentation (Heim et al., 2021, this issue). The results showed that CETA in its original form seems to be largely culture-sensitive for this target group. Mainly surface adaptations were made, especially for eCETA due to its asynchronous communication.

Based on our formative research, the cultural adaptation of the manual comprised three main aspects: i) cultural concepts of distress in the target population (i.e. Arabic speakers from the MENA region), ii) treatment components to address post-migration living conditions, and iii) treatment delivery, i.e., the provision of an additional online version to address potential treatment barriers.

Concerning the cultural concepts of distress, all adaptations are in line with previous findings. The qualitative interviews showed that the introduction of CETA should be expanded to include an explanatory model to address cultural explanations. Thus, the relationship between physical and mental well-being is now more clearly demonstrated and explained, since the literature underlines that Arabic idioms of distress do not distinguish between somatic experiences and psychological problems (Hassan et al., 2015). Furthermore, the “fear of going crazy” (Shannon, 2014) was addressed by explaining the concept of mental disorders and psychological treatment. To assess suicidal ideation, different opinions were expressed, which tended either to assess suicidal ideation directly or indirectly. This difference was affected both by culture (e.g. suicide is a crime in some Arab countries, Hassan et al., 2015) and by legal aspects of the German health care system (suicidality must be clearly clarified). Accordingly, the adaptation comprises the gradual assessment of suicidal tendencies (i.e. starting with an indirect question, followed by a direct question).

With regard to treatment components, the results indicated that the specific CETA components as well as the unspecific elements are suitable for the current context of Arabic-speaking refugees in Germany. This is in line with a review examining the effectiveness of psychological interventions in different low- and middle-income countries (Singla et al., 2017). The specific component of "Problem Solving" was considered to be important in the discussions of the research team and in the literature (Singla et al., 2017). The difficult living conditions, in which refugees have to deal with multiple social problems (e.g. asylum process, housing), have been shown to affect refugees’ mental health (Schick et al., 2018). To address these difficulties, “Problem Solving” will be offered to every patient in order to provide problem-solving skills to manage some of these existential problems.

With regard to treatment delivery, an online version of CETA was developed. Since this type of asynchronous communication requires more active patient involvement, the therapeutic tasks have to be described in more detail and include more examples. Thus, some adaptations will only be applied in eCETA. Adaptations with regard to materials mostly referred to analogies, as well as examples and translations of words or phrases. A distinction was made between linguistic adaptations (e.g. translation of the phrase "a day in the life") and adaptations based on culture and migration (e.g. typical receptacles used for alcohol, everyday situations). This is in line with other studies in the field (e.g., Shala et al., 2020).

In sum, a small number of mainly surface adaptations were required. This might be a consequence of the fact that CETA was developed particularly for culturally diverse groups, already used simple language, already had an easily understandable structure, and has been used in different countries (Murray et al., 2014). This very well thought-out structure of the original CETA provided an excellent basis for the current adaptation process.

Even though the cultural adaptation was facilitated by the existing framework, some limitations remain. First, only people from two different cities in Germany were interviewed, and most of them were from Syria. However, the interviewees were of different ages and gender, and the four Arabic-speaking experts were from different countries of origin. Second, although we did not consider the entire CETA manual for adaptation, we selected an exact choice of words to explain a technique, main parts, and all interventional components that corresponded to the framework (Heim & Kohrt, 2019). Third, the decision to use the online format with an asynchronous communication was made prior to the formative research. These decisions are based on known contextual conditions (e.g. fear of stigmatisation, difficulties in accessing the health care system). All further adjustments to the format were then again part of the formative research.

The conceptual framework and the RECAPT guidelines simplify, standardise and clarify the cultural adaptation process. It can thus be summarised that adaptations do not always have to start from scratch; rather, practitioners and researchers are able to use existing material. Future research needs to compare different levels of adaptation and their impact on treatment acceptance and effectiveness. Such results might enable a balance between adaptation and the required time and financial effort.

## Supplementary Materials

The Supplementary Materials (for access see Index of Supplementary Materials below) include detailed information about:

1. Components of CETA and decision regarding remaining, adaptation or exclusion from the adapted manual (Supplement 1)
2. Process of cultural adaptation based on the reporting criteria (RECAPT, Supplement 2)
3. Extract from the adaptation monitoring form (Supplement 3)
4. Qualitative Research Checklist (COREQ, Supplement 4)
5. Surface adaptations (Supplement 5)

10.23668/psycharchives.5137 Supplement 1 Supplementary materials to "From formative research to cultural adaptation of a face-to-face and internet-based cognitive-behavioural intervention for Arabic-speaking refugees in Germany"

BöttcheM.
KampisiouC.
StammelN.
El-Haj-MohamadR.
HeekeC.
BurchertS.
HeimE.
WagnerB.
RennebergB.
BöttcherJ.
GlaesmerH.
Gouzoulis-MayfrankE.
ZielasekJ.
KonnopkaA.
MurrayL.
KnaevelsrudC.
 (2021). Supplementary materials to "From formative research to cultural adaptation of a face-to-face and internet-based cognitive-behavioural intervention for Arabic-speaking refugees in Germany"
[Additional information]. PsychOpen. 10.23668/psycharchives.5137
PMC967082836405676
