# Supplementary material for: From Formative Research to Cultural Adaptation of a Face-to-Face and Internet-Based Cognitive-Behavioural Intervention for Arabic-Speaking Refugees in Germany
Source: Clin Psychol Eur. 2021 Nov 23;3(Spec Issue):e4623. doi: 10.32872/cpe.4623 (PMC9670828; doi:10.32872/cpe.4623)
Supplement: Supplement 1 [file cpe-03-4623-s01.pdf]

From formative research to cultural adaptation of a face-to-face and internet-based cognitive-behavioural intervention for Arabic-speaking refugees in Germany

Maria Böttche, Christina Kampisiou, Nadine Stammel, Rayan El-Haj-Mohamad, Carina Heeke, Sebastian Burchert, Eva Heim, Birgit Wagner, Babette Renneberg, Johanna Böttcher, Heide Glaesmer, Euphrosyne Gouzoulis-Mayfrank, Jürgen Zielasek, Alexander Konnopka, Laura Murray, Christine Knaevelsrud

Manuscript published in Clinical Psychology in Europe
https://doi.org/10.32872/cpe.4623

Supplement 1

Table 1: Components of CETA (based on Murray et al., 2014) and cultural adaptations

Component	Abbreviation	Short Description	Suitable for adapted manual Results from HE interviews (n=6)	Techniques excluded based on results from research group discussions	Remaining techniques based on AP interviews
Encouraging Participation	EP	• Attention to engagement	*	+	-
Introduction	INTRO	• Information about CETA,<br>• Normalizing/validating problems/symptoms	*	+	-
Thinking in a Different Way: Part I and Part II	TDW-I<br>TDW-II	• Association between thoughts, feelings and behaviour<br>• Evaluate and restructure thinking to be more functional	• Yes, 5/6	• TDW-II: logical questioning, providing facts	• TDW-I: usage of triangle<br>• TDW-II: responsibility cake, family/friend role play, list/definition
Talking about Difficult Memories	TDM	• Confronting with traumatic memories<br>• Gradual imaginal exposure	• Yes, 4/6	+	• Imaginal exposure
Getting Active	GA	• Pleasurable and positive activities	• Yes, 5/6	+	• Positive activities
Relaxation	RELAX	• Strategies to reduce physiological tension	• Yes, 5/6	• Imagination of a safe place; progressive muscle relaxation	• Breathing
Substance Use	SU	• Concepts of Motivational Interviewing<br>• Change substance-related behaviour	• Yes, 4/6	+	• New activities, carrying reminders, avoiding places, letting the urge pass, saying no
Live Exposure	LE	• In vivo exposure to triggers/reminders	• Yes, 5/6	+	• Exposure
Problem Solving	PS	• Defining a problem, evaluating solutions<br>• Creating steps to test the chosen solution	• Yes, 4/6	+	-
Safety	SAFETY	• Assessing risk of suicide, homicide and domestic violence<br>• Safety plan	• Yes, 5/6	+	-

Notes: * component was not part of the HE interviews; + all techniques remained in the adapted manual; - there are no specific techniques included in the component and therefore none were asked about in the AP interview

Supplement 2

For the process of cultural adaption, we followed the reporting criteria A-C of Heim, Mewes et al., under review. These criteria were used for the top-down cultural adaptation process of an existing intervention (CETA) and were used as a guide for the process of cultural adaptation.

**A) Set-up**
Criterion 1:<br>The target population is defined as adult Arabic-speaking refugees from the MENA region living in Germany
Criterion 2:<br>The team involved in the process of adaptation consists of the following groups:<br>1) Interviewer (freelancer and employed):<br>a) Free List Interview (Arabic users, AU): Two independent freelance Arabic-speaking interviewers (male/female) conducted and summarised 20 interviews (10 each). The participants were not known to the interviewers.<br>b) Key Informant Interview (Arabic professionals, AP): Two independent freelance Arabic-speaking interviewers (male/female) conducted and summarised the 11 interviews (5 and 6 interviews, respectively). Participants were known on a professional level but not related to the study.<br>c) Key Informant Interview (Health experts, HE): Six interviews were conducted by three female employed interviewers from the research team. Participants were known on a professional level.<br>d) Focus groups (FG): Two independent freelance Arabic-speaking interviewers (male/female) conducted and summarised the focus groups (male and female group). Participants were known on a professional level but not related to the study.<br><br>All interviewers had at least a Bachelor degree in psychology. All interviewers received special training in advance as well as a structured written guideline for the interview.<br>All participants received study information in which the research topic and the goals of the research were transparently explained.<br><br>2) Research team (employed): one female researcher with a PhD led the process of cultural adaptation. In addition, five female researchers with at least a Bachelor degree were part of the team (two PhD, two M.Sc, one B.Sc.). This team discussed and decided on all adaptations.<br><br>3) Independent Arabic-speaking experts (freelancer): These four persons were all native-speaking health experts and not part of the interviewer or research team. They all had at least a Bachelor degree in psychology. Two of them were male. Two of them were working with refugees in the MENA region. Two of the experts had no relationship to the research team, while the other two were not related to the study but known on a professional level. The experts were familiar with the research topic, and the goals of the research were transparent.

Criterion 3:<br>A reporting form for the process of adaptation as suggested by Heim, Mewes et al., under review was used for the documentation of the process. For a better understanding, a section of this form can be found under Supplement 3.
Criterion 4:<br>At the current stage, the process of cultural adaptation of the original CETA manual was realised with the help of formative research. The authors plan to adapt the intervention also "on the fly" in the pilot testing and randomised controlled trial.
**B) Formative research**
Criterion 5:<br>For the process of formative research, the cultural adaptation monitoring form was used (Heim, Mewes et al., under review) and underwent the following steps in this order:<br><br>1) Literature review of existing information on the target group with regard to e.g. main characteristics, symptoms, syndromes. The literature search should give first insight into the existing literature regarding the understanding of mental disorders, culturally influenced symptom descriptions and Arabic expressions for mental health-related problems, feelings and thoughts. The results were used to support the findings of the interviews.<br><br>2) Qualitative interviews were conducted including free list and key informant interviews and focus groups. The COREQ checklist for the reporting of the qualitative data is available in Supplement 4.<br><br>For the free list interviews (AU), a convenience sampling was used. Participants were approached face-to-face. All participants were Arabic native speakers. The participants were potential users without a professional medical or psychosocial background. The majority of them had a refugee background. The interviews lasted approx. 60 minutes. The information from all interviews was summarised quantitatively (see data analysis).<br>Open-ended questions of the interviews were as follows: stating typical (non-helpful) thoughts and feelings of refugees in Germany; stating typical daily stressful situations; naming barriers and challenges to mental health access; naming the impact of alcohol, drugs and gambling; naming positive activities; naming places to drink, take drugs and gamble; naming persons which whom someone drinks, takes drugs, gambles; providing thoughts on the explanation of trauma with the help of a metaphor.<br><br>Key informant interviews (AP): A purposive sampling was used. Participants were interviewed face-to-face or by telephone. Participants were Arabic-speaking mental health professionals with a migration or refugee background. The interviews lasted approx. 120 minutes. The information was summarised quantitatively (see data analysis).<br>Open-ended questions of the interviews were as follows: giving opinions about the feasibility of the main cognitive restructuring techniques, breathing exercises as well as worksheets for substance use; giving examples for the reduction of substance abuse, for pictures of common beverages, drugs and gambling as well as for stressful situations which are not dangerous; giving opinions about certain CETA skills to reduce substance use; were asked about how to address suicidality and what individuals can do in this situation and whom to contact; giving opinions about the description of psychotherapy and analogies of the importance of continuity.

Key informant interviews (HE): A purposive sampling was used. Participants were approached face-to-face or by telephone. The participants were mental health experts working with refuges in different organisations in Germany. The interviews lasted approx. 60 minutes. The information was summarised quantitatively (see data analysis).

Open-ended questions of the interviews were as follows: naming treatment techniques which are effective or challenging; giving opinions about the different CETA components (Supplement 1).

Focus groups: A purposive sampling was used. Due to COVID-19 restrictions, data collection was realised by video call. The participants were Arabic-speaking mental health professionals with a migration or refugee background and partly overlapped with the key informants (AP). The interviews lasted approx. 120 minutes. The aim of the focus group was to discuss and underpin the inconsistent findings from the free list and key informant interviews.

Open-ended questions of the interviews were as follows: opinions about the therapeutic rationale; discussion about non-helpful thoughts derived from the free list interviews as well as examples for cognitive restructuring; discussion about the component “problem solving”; discussion about the skill “letting the urge pass” in the component “Substance Use”; discussion about the involvement of the Imam in crisis situations.

All interviews were conducted in a bilateral setting in which only the interviewer and participant were present (except for the focus groups, in which more participants were present). All participants who were contacted agreed to participate in the study. There were no dropouts. After receiving detailed information about the study, participants signed an informed consent form prior to participating in the interviews/focus groups. All forms were provided in Arabic (AU and AP, and FG). All interviews were conducted once with the respective interviewee.

All interviews were semi-structured, i.e. all questions were formulated prior to the interview and the interviewer had to conduct the interview with the help of a guided interview protocol.

The interview was tested in advance during the special training for the interviewers. Here, the interview was checked for understanding as well as of choice of words (Arabic).

All interviews/focus groups were audio-recorded, and the recordings were summarised and translated into German. In addition, the interviewer also made notes during and after the interview if needed. Summarised interviews were not returned to participants for further comments.

All summaries were coded by the first author. All themes identified were derived from the data. The description of the coding tree is not provided and participants did not provide feedback on the findings, but the focus groups did.

Data saturation was discussed. A complete saturation was not achieved, since the main goal of the interview was to obtain the most important ideas and concepts. Therefore, we aimed to figure out the most salient concepts to be able to adapt the existing intervention. As Weller et al, 2018 stated, a small sample of n = 10 persons can collect some of the most salient themes. A sample of n = 20 is more sensitive (Weller et al., 2018).

3) Barts Explanatory Model Inventory-Checklist (BEMI-C, Rüdell, 2009) was used for quantitative data assessment. The BEMI-C was completed by the free list participants (AU) and key informant participants (AP). It includes four different lists with regard to symptoms, causes, consequences, and treatments of distress. The answers could be binary-coded (present/not present). A positive/present answer was added up for each item within the four lists, enabling a quantitative statement about which item was mentioned particularly frequently in each interviewed group. There are no cut-off scores for the evaluation. The reliability of the different lists was acceptable and the BEMI-C has good face, content and external validity (Rüdell, 2009). In this study, only a part of the BEMI-C was used (Table 2).
4) Group discussions of the research team. The first author provided the results of the coding which were the basis of the discussion. With the help of the cultural adaptation monitoring form (supplement 3), the results of the qualitative and quantitative methods were discussed and a potential adaptation was suggested.
5) Expert decisions. Four independent Arabic-speaking mental health experts living in Germany and in the MENA region commented on the decisions and adaptations of the research group. This process was also part of the monitoring form (supplement 3) and lasted approx. 90 minutes for each expert.
6) Group discussions of the research team. A final decision on the suggestions of the experts was made.

Criterion 6:<br>The importance of cultural concepts of distress (CCD) for cultural adaptation is prominent. CCD could be separated into five subcategories, which could be explained as follows for individuals living in the MENA region:<br>1) Core beliefs about human suffering, i.e. general assumptions about human suffering and healing. Fatalism (i.e. suffering is part of human life and has to be endured with patience) as well as fate seem to be core beliefs of people living in Arab countries (Hassan et al., 2015).<br>2) Mind-body concepts, i.e. explanatory models were found to combine somatic experiences and psychological symptoms because the two are interlinked (Hassan et al., 2015). In our study, we decided to extend the original introduction in order to have the opportunity to explain the rationale a little more (e.g. more detailed examples especially for refugees, detailed description of the treatment, addressing the fear of becoming crazy, stressing the relationship between body and soul, and raising awareness of the concept of mental disorders and treatment options)<br>3) Culturally salient symptoms, i.e. symptom patterns seem to be anger/aggression, withdrawal, pain and ikti'ab' (Hassan et al., 2015).<br>4) Disorder-specific assumptions/beliefs, i.e. negative and positive beliefs about symptoms/disorders might arise from the assumption that psychological disorders are a test from God, God's will, or a form of punishment for sins (Dardas et al., 2015).<br>5) Idioms of distress, i.e. socially acceptable terms for expressing distress seem to be, e.g., sudden fear, depression, helplessness (Hassan et al., 2015). In our study, symptoms which are common in refugees, according to the BEMI-C, are named as examples in the eCETA version and are also part of the face-to-face manual (to provide the therapist with examples if the patients are unable to come up with any descriptions).<br><br>In addition to the CCD, the cultural adaptation also includes specific needs of the target group as well as contextual variables. In this study, the difficult access to the mental health system is one contextual variable which is addressed by the additional usage of an internet-based version.<br>The internet-based version enables a low-threshold access to the health system. This reduces the fear of stigmatisation, simplifies access to the health system and bridges geographical distance. The results of the adaptation of contextual variables are also explained in the result section.
**C) Intervention adaptation**
Criterion 7:<br>Specific treatment elements are theoretically and empirically based components of the intervention and assumed mechanisms of action. CETA focuses on different common mental disorders to address a broader symptom spectrum. In the process of cultural adaptation, the following specific elements were identified in CETA sessions: behavioural activation in "Getting Active", "problem solving", "relaxation", "exposure (live and imaginal)", cognitive restructuring in "Thinking in a different Way Parts I and II", self-monitoring in "Substance Use", Motivational Interviewing in "Substance Use", as well as identifying affects, linking affects to events and identifying thoughts in all CETA sessions.
Criterion 8:<br>Unspecific elements are universal to therapy experiences and are used for engaging the patient or implementing the treatment. Here, the treatment rationale is important. In addition, psychoeducation, empathic and active listening, normalising, discussing advantages and barriers to treatment, collaboration and wording are parts of unspecific CETA elements. An example in our study is the explanation of therapy with the help of a "mountain path": "The path that we will follow in the next few weeks is like a path high on a mountain or a path through an area that is not so well known. On this path there will be parts that are still quite easy to master and others that require a lot of strength and are difficult, where you have to make an effort. There will be moments when you may feel like you want to turn back and not get to the finish line, or when you get

off track or just don't want to go on. This is all quite understandable and happens on a long road. I would like to encourage you to follow the path you have started now to the end and I will help you as much as I can. But only you alone can take the path and master the individual stages on it"<br><br>Therapeutic techniques are skills that the therapist/intervention implements during a session to deliver an element. CETA-specific therapeutic techniques are role plays, behavioural experiments, providing direct suggestions, giving praise, assigning homework, and setting goals.
Criterion 9:<br>Surface adaptations include the delivery format (face-to-face and internet-based) as well as materials such as text, illustrations and case examples. The translation of certain words (e.g. "suicide" or "here and now") as well as metaphors (e.g. to explain a traumatic event) seems to be important for a cultural adaptation and therefore necessary to document. For some translations, the differentiation between adaptation and translation is not possible.

References

Dardas L. A., & Simmons, L. A. (2015). The stigma of mental illness in Arab families: a concept analysis. Journal of psychiatric and mental health nursing, 22(9), 668-679.

Hassan, G., Kirmayer, L. J., Mekki-Berrada, A., Quosh, C., El Chammay, R., Deville-Stoetzel, J. B., ... & Ventevogel, P. (2015). Culture, context and the mental health and psychosocial wellbeing of Syrians: A review for mental health and psychosocial support staff working with Syrians affected by armed conflict. Geneva: UNHCR, 13.

Heim, E., Mewes, R., Abi Ramia, J., Carswell, K., Hall, B. J., Harper Shehadeh, M., Glaesmer, H., Inçe Ünlü, B., Lechner-Meichsner, F., Lotzin, A., Moro, M. R., Salamanca-Sanabria, A., Singla, D. R., Starck, A., Sturm, G., Tol, W. A., Weise, C., & Verdeli, H. (2020). REporting Cultural Adaptation in Psychological Trials (RECAPT): List of criteria (Manuscript submitted for publication)

Rüdell, K., Bhui, K., & Priebe, S. (2009). Concept, development and application of a new mixed method assessment of cultural variations in illness perceptions: Barts Explanatory Model Inventory. Journal of health psychology, 14(2), 336-347

Weller, S. C., Vickers, B., Bernard, H. R., Blackburn, A. M., Borgatti, S., Gravlee, C. C., & Johnson, J. C. (2018). Open-ended interview questions and saturation. PloS one, 13(6), e0198606.

Supplement 3

Decision No.	Treatment components / delivery	Content / intervention	Translation / Adaptation	Evidence base<br><br>e.g., literature review, focus groups, qualitative interview	Quality of evidence<br><br>Strong<br>Moderate<br>Weak	Suggestions from research team				State of decision	
						Researcher 1 (PS)	Researcher 2 (AH)	Researcher 3 (JAR)	Researcher 4 (PN)	pending	made
1.0<br>CETA & eCETA	Specific factors	Specific factors in CETA<br><br>All specific factors are suitable for the context (refugees in Germany).<br><br>CETA consists of different sessions which focus on different problems<br><br>Sessions are:<br>• Cognitive restructuring "Think in a different way, TDW"<br>• In Sensu Exposure "Talking about difficult memories, TDM"<br>• "Relaxation; RELAX"<br>• Positive activities "Getting active, GA"<br>• Dealing with substance abuse "Substance use reduction, SU"<br>• In-Vivo Exposure "Life exposure"<br>• Problem Management "Solving Problems, SM"<br>• Dealing with suicidal ideation, homicidal ideation, domestic violence "safety"	All sessions seem to be appropriate for refugees. Key Informants as well as professionals use these techniques in their work with refugees or find these sessions/techniques suitable.<br><br>No changes are made	Qualitative interviews	strong	Suggestion<br>Informational module on COVID-19 and topics related to migration (asylum, family reunion, etc.)	agree	Agree- note: problem management was considered challenging for internet-based interventions with refugees in Lebanon. Further guidance from professionals needs to be ensured.<br>We ended up replacing this with gratitude exercises and focused on techniques related to: behavioural activation, stress management, positive self-talk, garnering social support, and relapse prevention	Agree – to keep in mind that alcohol consumption is considered a sin/'haram' to some, important to be sensitive when asking questions related to alcohol abuse.		x
1<br>CETA & eCETA	Mind-body concepts	Additional parts in the sessions "Encouraging Participation" and "Introduction"<br><br>These two sessions are the first sessions with which CETA starts.<br><br>It seems to be important to have a rationale at the beginning of the treatment to explain, and avoid dropout directly at the beginning, especially for the internet-based version.	The formative research underlined that the following idioms are especially important to address:<br>• Fear of becoming crazy<br>• Relationship between body and soul<br>• Awareness of concept of mental disorders and treatment options<br><br>We decided to extend the original introduction to have the opportunity to explain the rationale a little more (e.g. slightly more detailed examples especially for refugees, detailed description of the treatment as a kind of path).<br>This section is not entirely included in CETA but parts of the questions are already included.<br>Please see document *EP_INTRO_rev6* "**rationale and anamnesis**" & "**Description CETA and treatment**"<br><br>We adapted the original CETA	Literature review<br>Qualitative interview<br>Focus group	strong	Suggestion<br><br>Check the corresponding document "*EP_INTRO_rev6*".	agree	Agree.<br>Quick notes about EP intro rev4:<br>-Ask explicitly: What brought you here? (and not just about symptoms or problems)<br>-Ask beneficiaries about their expectations of this programme, before explaining to them what it entails<br>-Try not to be overly assertive when talking about the results/effectiveness of the programme. Giving too much	Added my comments to the document.		x

								hope might create very high expectations and can be tricky.<br>Suggestion to replace "this will help you → with: this might/can help you			
2<br>CETA & eCETA	Specific factor	Session "Problem management"<br><br>Qualitative interviews with health professionals showed that dealing with unsolvable problems as well as emotion regulation is an important factor for working with refugees.	At first, we decided to develop a new module for these kinds of unsolvable problems. We did not want to develop an emotion regulation module because this is not directly in line with the principles of CETA and we focused on approaches with regard to acceptance.<br>We discussed different versions of this module (experts as well as in focus groups). After several discussions, we decided that unsolvable problems should be treated with the already existing module "Thinking in a Different Way" (restructuring of thoughts to change behaviour and emotions).<br>Therefore, the already existing module of "Problem Management" will remain in its original format and will not get additional parts.<br><br>No changes are made	Qualitative interviews, Focus groups, Expert Interviews	strong	Agree to deal with it in the module cognitive restructuring.<br><br>Suggestion: Acceptance can be part of the module relaxation	agree	Agree	I suggest adding this point in Intro/EP when introducing TDW in Intro/EP: The therapist might not be able to help you change your reality but might be able to help you cope better with it by changing your perspective through TDW.		X
3<br>CETA & eCETA	Specific factor	Session "Problem management, PM"<br><br>The session "PM" is only used in original CETA when there is a specific problem named by the client or indicated by a questionnaire.	Due to cohort-specific post-migration problems, all clients will receive a PM module at the end of the treatment (i.e. after they finished all the symptom-specific sessions)<br><br>We did not change the session "Problem management" but all clients will receive this session<br><br>No changes are made	Qualitative interviews<br>Focus groups<br>Expert Interviews	strong	Agree	agree	Agree	Agree that everyone should learn PS.		x
4<br>CETA & eCETA	Surface	Using logos of the universities in certain official papers (e.g. informed consent)	It seems to be more trustworthy to mention the participating partners, and therefore, they will be added in certain official documents<br><br>No changes are made	Qualitative interviews	strong	Agree	agree	Agree	Agree		x
6<br>CETA & eCETA	surface	Session "Encouraging Participation"<br><br>We wanted to know if mentioning that the treatment is based on research is well received or might create some ambivalent feelings because research sometimes seems to have an ambivalent connotation: Sentence in CETA: "Research on the program indicates that it works. This program has helped many people around the world to feel better and have less distress"	Mentioning that the treatment components are based on research as it is described in CETA seems to be more trustworthy, and therefore, the sentence remains in the manual:<br><br>No changes are made	Qualitative interviews	strong	Suggestion:<br><br>Agree to mention research and evidence. Also important that the therapist emphasises the personal experience of having helped many people (suffering from similar problems) to get better and overcome.	agree	Agree	Agree to keep it and to say that people around the world feel better…		x
7<br>CETA & eCETA	Unspecific factor	Session "Encouraging Participation"<br><br>Due to the fact that the majority of refugees are not familiar with the concept of psychotherapy, we wanted to include an analogy to describe the process of treatment.	The analogy has to be clear as well as the understanding of the different stages in the treatment (ups and downs, hard work for client, has to go on his/her own). We included the following description:	Qualitative interviews<br>Literature review<br>Expert discussion	strong	Suggestion<br><br>Please check changes to the Arabic translation.	إن الطريق الذي سوف نتبعه في الأسابيع القليلة المقبلة يشبه طريقاً مرتفعًا على جبل أو طريقاً عبر	Agree with the analogy- yet, I suggest to remove "a path through an area that is not well known" because	Agree and suggest also mentioning that therapy consists of work in session but also of practising what they learn at home.		x

		In CETA, the description of the sessions is explained as a journey on the mountain/pathway.<br>In the manual, no explicit phrasing of this journey/pathway is given.	*"The path that we will follow in the next few weeks is like a path high on a mountain or a path through an area that is not so well known. On this path there will be parts that are still quite easy to master and others that require a lot of strength and are difficult, where you have to make an effort. There will be moments when you may feel like you want to turn back and not get to the finish line, or when you get off track or just don't want to go on. This is all quite understandable and happens on a long road. I would like to encourage you to follow the path you have started now to the end and I will help you as much as I can. But only you alone can take the path and master the individual stages on it"*<br><br>إن الطريقة الذي سوف نتبعه في الأسابيع القليلة المقبلة يشبه طريقاً مرتفعًا على جبل أو طريقاً عبر منطقة غير معروفة جيدًا. على هذا الطريق سيكون أجزاء سهلة وغيرها تتطلب كثيراً من القوة وصعبة، حيث عليك أن تبذل جهدا. ستكون في لحظات عندما قد تشعرأن تريد أن ترجع و لم تصل إلى خط النهاية، أو إذا ابتعدت عن الطريق أو لا تريد أن تستمر. هذا هو كل شيء مفهوم تماما ويحدث على طريق طويل. أود أن أشجعك على اتباع الطريقة التي بدأت الآن حتى النهاية وسأساعدك قدر ما أستطيع. ولكن فقط أنت لوحدك يمكن أن تمشي الطريق وإتقان المراحل الفردية.<br><br>We adapted the original CETA<br><br>FINAL DECISION (after additional expert team discussion (CW, CKa, CH, NS, SP, MB, RM, LH)):<br>*"The path that we will follow in the next few weeks is like a path high on a mountain. The aim is to reach the top of the mountain and CETA will enable you to have the skills to do that. On this path of improvement there will be parts that are quite easy to master and others that require a lot of strength and are difficult, where you have to make an effort. There will be moments when you may feel like you want to turn back and not get to the finish line, or when you get off track or just don't want to go on. This is all quite understandable and happens on a long road. I would like to encourage you to follow the path you have started now to the end and I will help you as much as I can along the way. But only you alone can take the path and master the individual stages on it"*<br>إن الطريق الذي سوف نتبعه في الأسابيع القليلة المقبلة يشبه طريقاً مرتفعًا على جبل. الهدف هو الوصول إلى قمة الجبل وستمكنك سيته من امتلاك المهارات اللازمة للقيام بذلك. على هذا طريق التحسين ستكون هناك أجزاء سهلة وأخرى صعبة وتتطلب الكثير من القوة، حيث عليك أن تبذل جهدا. ستكون في لحظات عندما قد تشعر أن تريد أن ترجع و لم تصل إلى خط النهاية، أو إذا ابتعدت عن الطريق أو لا تريد أن تستمر. هذا أمر مفهوم تماما ومن الطبيعي أن يحصل على طريق طويل. أود أن أشجعك على اتباع الطريق الذي بدأته الآن حتى النهاية وسأساعدك على طول الطريق بقدر ما أستطيع. ولكن فقط أنت لوحدك يمكن أن تمشي الطريق وإتقان المراحل الفردية.			إن الطريق الذي سوف نتبعه في الأسابيع القليلة المقبلة يشبه طريقاً مرتفعًا على جبل أو طريقاً عبر منطقة غير مألوفة جيدًا. على هذا الطريق سيتكون هناك أجزاء سهلة وأخرى صعبة وتتطلب الكثير من القوة، حيث عليك أن تبذل جهدا كبيراً. ستكون هناك لحظات قد تشعر فيها أنك تريد أن ترجع وتشعر أنك لن تصل إلى خط النهاية، أو تشعر أنك ابتعدت عن الطريق أو أنك لا تريد الاستمرار. هذا أمر مفهوم تماما ومن الطبيعي أن يحصل على طريق طويل. أود أن أشجعك على اتباع الطريق الذي بدأته الآن حتى النهاية وسأساعدك قدر ما أستطيع. ولكن فقط أنت وحدك عليه أن يسير ويتقن كل مرحلة على هذا الطريق.	منطقة غير معروفة جيدًا. على هذا الطريق سيكون أجزاء سهلة وغيرها تتطلب كثيراً من القوة وصعبة، حيث عليك أن تبذل جهدا. ستكون في لحظات عندما قد تشعرأن تريد أن ترجع و لم تصل إلى خط النهاية، أو إذا ابتعدت عن الطريق أو لا تريد أن تستمر. هذا ~~هوكل~~ شيء مفهوم تماما ويحدث على طريق طويل. أود أن أشجعك على اتباع الطريق التي بدأت الآن حتى النهاية وسأساعدك قدر ما أستطيع. ولكن فقط أنت لوحدك يمكن أن تمشي الطريق وإتقان المراحل الفردية.<br><br>Without: هو كل	it creates confusion, and it's better for the beneficiaries to stay focused on one example, the one of the mountain.<br><br>Also, the Arabic translation needs to be carefully and substantially revised. Please refrain from using google translate and make sure someone proofreads it. There are many grammatical and structural mistakes.	I suggest adding in the analogy that the path is one that leads to improvement; the path of improvement/the path to healing.		
8<br>**eCETA**	Culturally salient symptoms, Idioms of distress	Session "Encouraging Participation"<br><br>In this session, the therapist says: "this program teaches skills to improve feelings of (*name client's symptoms*)."	Due to the fact that in the eCETA version, there is no simultaneous communication and the client has to undergo the session on his/her own, the client needs a more detailed description of the task but also some examples to know what possible answers might be.<br><br>Therefore, frequent symptoms which are common in refugees are named as examples in the eCETA version. These symptoms will be filled in the text in EP for eCETA :<br>"*This program teaches skills to improve feelings of:*<br>• *Sleeping problems*<br>• *Anxiety and fear*<br>• *Concentration problems*	Literature review<br>BEMI-C<br>Expert discussion	strong	The Arabic translation does not match the English text.<br>See corrections<br><br>مشاكل النوم<br>القلق والخوف.<br>مشاكل في التركيز<br>الإرهاق/ التعب.<br>فقدان الراحة.<br>æ<br>"مشاكل في النوم والتركيز"	agree	Agree-<br>Note:<br>Put:<br>This program teaches skills to improve the following (instead of feelings because sleeping problems are concentration problems and not feelings).<br>Also, put restlessness	Agree		x

			• *Fatigue/tiredness*<br><br>*and Sleeping problems and Concentration problems"*<br><br>هذا البرنامج يعلم المهارات اللازمة لتحسين مشاعر:<br>. القلق والخوف<br>. التعب/ النعس<br>. هائج<br>æ<br>مشاكل في النوم<br>æ<br>مشاكل في التركيز<br><br>We adapted the original CETA (only for eCETA)<br>In the face-to-face sessions, the symptoms will be figured out together with the client<br><br>FINAL DECISION (after additional expert team discussion):<br>"*This program teaches skills to improve problems with:*<br>• *Sleeping problems*<br>• *Anxiety, fear and stress*<br>• *Concentration problems*<br>• *Fatigue/tiredness*<br>• *Stressful memories*<br>• *Sadness"*<br><br>هذا البرنامج يعلم المهارات اللازمة لتحسين المشاكل مع :<br>. المشاكل في النوم<br>. القلق والخوف و التوتر<br>. المشاكل في التركيز<br>. التعب/ النعس<br>.ذكريات مرهقة<br>. الحزن					instead of restless, to stay consistent and adapt the Arabic translation too.<br>Arabic translation does not match the English one; it should be:<br><br>هذا البرنامج يعلم المهارات اللازمة لتحسين مشاعر:<br>- المشاكل في النوم<br>. القلق والخوف<br>- المشاكل في التركيز<br>. التعب<br>. الهيجان<br><br>and *Sleeping problems and Concentration problems"* is already mentioned in the bullet points so better remove it in English and Arabic			
9<br>**eCETA**	surface	Session "Encouraging Participation"<br><br>This session also deals with unhelpful thoughts which are named by the therapist to explain which problems CETA can help with.<br><br>In the original CETA, the thoughts will be figured out together with the client:<br>*"We also may say things to ourselves in our heads or think in a certain way about past events and things in life that make us feel angry, sad or worried. For examples (add an unhelpful thought they may have)".*<br><br>.	Due to the fact that in the eCETA version, there is no simultaneous communication and the client has to undergo the session on his/her own, the client needs a more detailed description and the thoughts have to addressed directly<br>The qualitative research resulted in the following examples for non-helpful thoughts in refugees. These examples will be used to demonstrate possible unhelpful thoughts.<br><br>*"We also may say things to ourselves in our heads, or think in a certain way about past events and things in life that make us feel angry, sad, or worried. For example,*<br>*"My life will not be the same",*<br>*"I have the feeling that I have not found my role/place in German society",*<br>*"I am worried about my residence permit"*<br><br>يمكننا أيضًا أن نقول لأنفسنا أشياء في رؤوسنا ، أو نفكر بطريقة معينة في الأحداث الماضية وأشياء في الحياة تجعلنا نشعر بالغضب أو الحزن أو القلق, مثلاً:<br><br>لن تعود حياتي كما كانت من قبل.<br>.<br>لا اشعر ان لي مكان في المجتمع الالماني<br>أنا متوتر بشأن تصريح الإقامة.<br><br>We adapted the original CETA (only for eCETA)	Qualitative interviews<br>Focus groups<br>Expert discussion	strong	Suggestion:<br><br>Worrying about the residence permit is realistic and valid (at least for many refugees).<br><br>يمكننا أيضًا أن نقول لأنفسنا أشياء في رؤوسنا، أو نفكر في الأحداث الماضية وأشياء في الحياة بطريقة معينة تجعلنا نشعر بالغضب أو الحزن أو القلق. مثلاً:<br><br>لن تعود حياتي كما كانت من قبل.<br>.<br>لدي شعور بأني لن أجد لي مكان في المجتمع الالماني<br>أنا متوتر بشأن تصريح الإقامة.	Instead of<br><br>في الأحداث<br><br>Preferably<br><br>بالاحداث	agree	I suggest the following thoughts:<br>"I will never be well again"<br>"I will not be able to get my residence permit"<br>"I don't think I will fit into the German society"<br><br>The above formulations would be easier when teaching TDW.		x

			In the face-to-face sessions, the thoughts will be figured out together with the client<br><br>FINAL DECISION (after additional expert team discussion)<br>*"We also may say things to ourselves in our heads, or think in a certain way about past events and things in life that make us feel angry, sad, or worried. For example,*<br>*"My life will not be the same",*<br>*" I will never be well again"*<br>*"I do not think I will fit into German society",*<br>*"I will not be able to get my residence permit"*<br><br>يمكننا أيضًا أن نقول لأنفسنا أشياء في رؤوسنا ، أو نفكر بطريقة معينة في الأحداث الماضية وأشياء في الحياة تجعلنا نشعر بالغضب أو الحزن أو القلق, مثلاً:<br><br>" لن تعود حياتي كما كانت من قبل"<br>"لن أكون بصحة جيدة ابدا"<br>"لا أعتقد أنني سأكون مناسبًا في المجتمع الالماني"<br>" لن أتمكن من الحصول على تصريح الإقامة."								
10<br>**eCETA**	surface	Session "Encouraging Participation"<br>In this session, clients have to name potential barriers/problems to engage in this treatment.<br><br>In the original CETA, the therapist addresses this issue if the client raises concerns. The manual offers the following examples:<br>*"I hear these concerns often. Sometimes people think this will not be helpful, it is too much time, or the family will not allow them to go."*	Due to the fact that in the eCETA version, there is no simultaneous communication and the client has to undergo the session on his/her own, the client needs a more detailed description and the concerns have to addressed directly.<br><br>We changed the examples in the manual for possible challenges and barriers that may jeopardise continuing the treatment as follows:<br>"*Sometimes, people have concerns about the treatment or there are barriers to why participation is difficult. Sometimes it is a fear of stigmatisation (e.g. no one will speak with me, my family and friends will turn away) or there is no or little trust in treatment and methods (this will not be helpful). And sometimes there is no private space or no possibility for childcare. Maybe you also have a barrier or a concern.*"<br><br>في بعض الأحيان الناس يشكون من العلاج أو يجدوا عَائِق, لذلك المشاركة صعبة. في بعض الأحيان يكون الخوف من الوصم (مثلاً: لا أحد يريد أن يتحدث معي, ستبتعد عائلتي و أصدقائي عني) او لا توجد ثقة أو ثقة قليلة في العلاج و الطريق (هذا لن يكون مفيدا).وفي بعض الأحيان لا يوجد مكان للخصوصية او لا توجد إمكانية لرعاية الأطفال. ممكن لديك عَائِق أو شك أيضًا.<br><br>We adapted the original CETA (only for eCETA)<br>In the face-to-face sessions, the challenges will be figured out together with the client<br><br>FINAL DECISION (after additional expert team discussion)<br>"*Sometimes, people have concerns about the treatment or there are barriers why a participation is difficult. Sometimes it is fear (e.g.* people will say I am crazy) *or there is no or little trust in treatment and methods (this will not be helpful). And sometimes there is no private space or no possibility for childcare. Maybe you also have a barrier or a concern*?"<br><br>في بعض الأحيان الناس يشكون من العلاج أو يجدون عوائق, مما يجعل المشاركة صعبة.<br>في بعض الأحيان يكون هناك خوف من الوصم (مثلاً: سيقول الناس إنني مجنون) أو لا توجد ثقة أو تكون هناك ثقة قليلة في العلاج و تقنياته(هذا لن يكون مفيدا).وفي بعض الأحيان لا يوجد مكان للخصوصية او لا توجد إمكانية لرعاية الأطفال. هل لديك أيضاً عَائِق أو شك؟	Qualitative interviews, Expert discussion,	strong	Check proof of the translation<br><br>أحياناً تكون لدى البعض تحفظّات على العلاج أو تكون هناك أمور تجعل المشاركة في العلاج صعبة. في بعض الأحيان يكون هناك خوف من الوصم (مثلاً: لا أحد يريد أن يتحدث معي، ستبتعد عائلتي و أصدقائي عني) أو لا توجد ثقة أو تكون هناك ثقة قليلة في العلاج و تقنياته(هذا لن يكون مفيدا).وفي بعض الأحيان لا يوجد مكان للخصوصية او لا توجد إمكانية لرعاية الأطفال. هل لديك أيضاً عَائِق أو شك؟.	Agree	I suggest putting the barriers in bullet points like the examples above.<br>The sentence below is not very clear and can be rephrased:<br>"*Sometimes, people have concerns about the treatment or there are barriers why participation is difficult.*<br>Better be more specific: ex: Sometimes, people don't trust the effectiveness of the treatment, or find logistical difficulties in abiding by it.<br><br>Also, in the brackets next to fear of stigmatisation, you can add the example of "they will think I'm crazy" as this is a major and common fear pertaining to mental health among refugees<br><br>As for the Arabic translation, it seems to be done on google translate. It doesn't read well.	I suggest to replace the sentence "no one will speak to me" to "people will say I am crazy"		x

								في بعض الأحيان الناس يشكون من العلاج أو يجدون عوائق, مما يجعل المشاركة صعبة. في بعض الأحيان يكون الخوف من الوصمة (مثلاً: لا أحد يريد أن يتحدث معي, ستبتعد عائلتي و أصدقائي عني) او انعدام الثقة أو ثقة قليلة في العلاج و المسار المتبع (هذا لن يكون مفيدا).وفي بعض الأحيان لا يوجد مكان للخصوصية او لا توجد إمكانية لرعاية الأطفال أثناء تلقي العلاج. ممكن أن يكون لديك عائق أو شكوك أيضًا.			
11<br>CETA & eCETA	surface	Session "Introduction"<br><br>The CETA manual uses the analogy of "going to school" or "taking antibiotics" to explain the importance of meeting regularly.<br><br>*"When you take an antibiotic, and you want it to work, can you take it only a few times, and take it whenever you want?*<br>*When children go to school, can they go only some days, whenever they want and still get their qualifications?*<br>*This program is like this story; we need to meet regularly, each week, for this to work*."	These examples seem to be appropriate and we will use CETA-conform examples. First the analogy of the antibiotics will be displayed. If the context is clear, the second will not be used. If it is unclear, the second analogy will be presented.<br><br>إذا كنت تتناول مضادًا حيويًا وتريد أن تعمل هل يمكنك أن تأخذه عدة مرات فقط و تأخذه في اي وقت تريد؟ عندما يذهب الأطفال إلى المدرسة ، هل يمكنهم الذهاب بعض الأيام فقط متى يريدون و رغم ذلك يحصلون على شهادتهم؟<br>هذا البرنامج مثل هذه القصة. نود ان نلتقي بانتظام كل أسبوع لكي ينجح هذا العلاج.<br><br>No changes are made	Qualitative interviews	strong	Check proof of the translation<br><br>ذا كنت تتناول مضادًا حيويًا وتريد أن تعمل هل يمكنك أن تأخذه عدة مرات فقط و تأخذه في اي وقت تريد؟<br>عندما يذهب الأطفال إلى المدرسة ، هل يمكنهم الذهاب بعض الأيام فقط متى يريدون و رغم ذلك يحصلون على شهادتهم؟<br>هل يستطيع الطلاب الحصول على شهاداتهم حتى لو ذهبوا إلى المدرسة لبضعة أيام فقط حسب الرغبة؟<br>هذا البرنامج مثل هذه القصة. نود ان نلتقي بانتظام كل أسبوع لكي ينجح هذا العلاج.	agree	Agree- great examples.<br>One additional analogy suggested is exercising/going to gym.<br>People tend to associate easily with progress related to physical activity, and some campaigns use the following motto "just like you take care of your body shape/or physical health, you can also train your mental health"	Agree		x
12<br>CETA & eCETA	In-session techniques	Session "Thinking in a Different Way I"<br><br>For the understanding of how thoughts are connected with feelings and behaviour and how changing a thought of the same situation changes the feelings and the behaviour, CETA uses triangles to describe the relationship (thought, feeling, behaviour):<br>*"We do not always have control over situations, but we can evaluate how we think about a situation, and change feelings and behaviours by thinking in a different way. There are skills you can learn to think differently about these unhelpful thoughts, so that you feel better*"	The formative research concluded that the general understanding of the triangle and the its explanation is suitable.<br>With a given example the triangle is comprehensible as it is described in CETA:<br><br>احيانا ما كل شىء تحت سيطرتنا, ولكن يمكننا أحيانا مراجعة طريقة تفكيرنا حول موقف ما وبالتالي تغيير المشاعر والتصرفات إذا فكرنا في طريقة مختلفة. يوجد مهارات فيك تتعلمها للتفكير بشكل مختلف في هذه الأفكار الغير مفيدين لكي تشعر بتحسن.<br><br>No changes are made	Qualitative interviews	strong	Check proof of the translation<br><br>احيانا ما كل شىء تحت سيطرتنا, ولكن يمكننا أحيانا مراجعة طريقة تفكيرنا حول موقف ما وبالتالي تغيير المشاعر التصرفات إذا فكرنا في طريقة مختلفة. يوجد مهارات فيك تتعلمها للتفكير بشكل مختلف في هذه الأفكار الغير مفيدين لكي تشعر بتحسن.	المشاعرو التصرفات<br><br>*There are skills you can learn to think differently about these unhelpful thoughts, so that you feel better"*<br><br>هنالك مهارات لتتعلم التفكير بطريقة مختلفة عن الافكار السلبية, لتجعلك تشعر بتحسن	Agree-<br><br>But the Arabic translation is in spoken dialect, whereas all the above is in the formal dialect (fos7a). Better be consistent.	Agree		x

Supplement 4

COREQ (COnsolidated criteria for REporting Qualitative research) Checklist

A checklist of items that should be included in reports of qualitative research. You must report the page number in your manuscript where you consider each of the items listed in this checklist. If you have not included this information, either revise your manuscript accordingly before submitting or note N/A

Topic	Item No.	Guide Questions/Description	Reported on Page No.
**Domain 1: Research team and reflexivity**			
Personal characteristics			
Interviewer/facilitator	1	Which author/s conducted the interview or focus group?	suppl. 2, Criterion 2
Credentials	2	What were the researcher's credentials? E.g. PhD, MD	suppl. 2, Criterion 2
Occupation	3	What was their occupation at the time of the study?	suppl. 2, Criterion 2
Gender	4	Was the researcher male or female?	suppl. 2, Criterion 2
Experience and training	5	What experience or training did the researcher have?	suppl. 2, Criterion 2
Relationship with participants			
Relationship established	6	Was a relationship established prior to study commencement?	suppl. 2, Criterion 2
Participant knowledge of the interviewer	7	What did the participants know about the researcher? e.g. personal goals, reasons for doing the research	suppl. 2, Criterion 2
Interviewer characteristics	8	What characteristics were reported about the inter viewer/facilitator? e.g. Bias, assumptions, reasons and interests in the research topic	suppl. 2, Criterion 2
**Domain 2: Study design**			
Theoretical framework			
Methodological orientation and Theory	9	What methodological orientation was stated to underpin the study? e.g. grounded theory, discourse analysis, ethnography, phenomenology, content analysis	Data Analysis
Participant selection			
Sampling	10	How were participants selected? e.g. purposive, convenience, consecutive, snowball	suppl. 2, Criterion 5
Method of approach	11	How were participants approached? e.g. face-to-face, telephone, mail, email	suppl. 2, Criterion 5
Sample size	12	How many participants were in the study?	Table 1
Non-participation	13	How many people refused to participate or dropped out? Reasons?	suppl. 2, Criterion 5
Setting			
Setting of data collection	14	Where was the data collected? e.g. home, clinic, workplace	suppl. 2, Criterion 5
Presence of non-participants	15	Was anyone else present besides the participants and researchers?	suppl. 2, Criterion 5
Description of sample	16	What are the important characteristics of the sample? e.g. demographic data, date	suppl. 2, Table 1
Data collection			
Interview guide	17	Were questions, prompts, guides provided by the authors? Was it pilot tested?	suppl. 2, Criterion 2
Repeat interviews	18	Were repeat inter views carried out? If yes, how many?	suppl. 2, Criterion 5
Audio/visual recording	19	Did the research use audio or visual recording to collect the data?	data collection
Field notes	20	Were field notes made during and/or after the inter view or focus group?	suppl. 2, Criterion 5
Duration	21	What was the duration of the inter views or focus group?	suppl. 2, Criterion 5
Data saturation	22	Was data saturation discussed?	suppl. 2, Criterion 5
Transcripts returned	23	Were transcripts returned to participants for comment and/or correction?	suppl. 2, Criterion 5

Topic	Item No.	Guide Questions/Description	Reported on Page No.
**Domain 3: analysis and findings**			
Data analysis			
Number of data coders	24	How many data coders coded the data?	suppl. 2, Criterion 5
Description of the coding tree	25	Did authors provide a description of the coding tree?	suppl. 2, Criterion 5
Derivation of themes	26	Were themes identified in advance or derived from the data?	suppl. 2, Criterion 5
Software	27	What software, if applicable, was used to manage the data?	data analysis
Participant checking	28	Did participants provide feedback on the findings?	suppl. 2, Criterion 5
Reporting			
Quotations presented	29	Were participant quotations presented to illustrate the themes/findings? Was each quotation identified? e.g. participant number	no
Data and findings consistent	30	Was there consistency between the data presented and the findings?	Results, yes
Clarity of major themes	31	Were major themes clearly presented in the findings?	Results, yes
Clarity of minor themes	32	Is there a description of diverse cases or discussion of minor themes?	Results, yes

Developed from: Tong A, Sainsbury P, Craig J. Consolidated criteria for reporting qualitative research (COREQ): a 32-item checklist for interviews and focus groups. *International Journal for Quality in Health Care*. 2007. Volume 19, Number 6: pp. 349 – 357

Supplement 5

CETA and eCETA		eCETA	
**Component**	**Selection of added examples in the manual (n out of x interviews, n/x)**	**Component**	**Selection of added examples in the manual (n out of x interviews, n/x)**
Exposure in vivo: Fearful situations	● Ride a bike (5/11)<br>● Drive a car (3/11)<br>● Learn to swim (2/11)	EP: Unhelpful thoughts	● Life will not be the same[a]<br>● I have not found my role/place in Germany[a]
SU: Images of alcohol and drugs	● Bottle of beer (7/11)<br>● Glass of wine (8/11)<br>● Cocaine (6/11)	TDW-I: Everyday situation	● Being in a place with a lot of people (11/20)<br>● Situations in which I have to speak German (8/20)
SU: Effects due to consumption	● Financial problems (16/20)<br>● Health problems (10/20)<br>● Problems with family (6/20)	TDW-I: Feelings	All examples can be found in Table 2
SU: Places of consumption and persons with whom one consumes	Alcohol<br>● Bars (10/20)<br>● Streets (8/20)<br>Drugs<br>● Bars (7/20)<br>● Clubs (5/20)<br>Friends (15/20)<br>Strangers (6/20)	GA: Positive activities	● Sport, e.g. swimming (18/20)<br>● Social activities, e.g. cooking with friends (9/20)<br>● Nature: park, garden, woods (10/20)
SU: ABC scheme	Existing examples were slightly adapted	TDW-II: Dysfunctional thoughts	● I don't feel part of society[a]<br>● Life will not be the same[a]
SU: Carrying a reminder	● Picture of children or mother (5/11)	EP: Barriers to therapy	● Fear of stigmatisation (15/20)<br>● No or little trust in treatment and methods (8/20)
		SU: Finding new activities	● Sport, e.g. swimming (9/20)<br>● Hobby (6/20)
		Safety: Helpful things to decrease thoughts	● Talking to someone (6/11)<br>● Not being alone (5/11)<br>● Being aware of the thought (5/11)
		Safety: People you can talk to	● Friends (7/11)<br>● Professionals (3/11)
		Finish: Celebrate the end	● Celebration with family/friends (10/20)<br>● Meeting friends (8/20)

Notes: SU Substance Use; EP Encouraging Participation; TDW Thinking in a Different Way, GA Getting Active.

(n/11) refers to AP, (n/20) refers to AU interviews. *n* refers to the number of participants who named the example.

[a] examples are discussed and the wording was slightly changed by the focus groups
